# Accuracy and Repeatability of Spatiotemporal Gait Parameters Measured with an Inertial Measurement Unit

**DOI:** 10.3390/jcm10091804

**Published:** 2021-04-21

**Authors:** Jorge Posada-Ordax, Julia Cosin-Matamoros, Marta Elena Losa-Iglesias, Ricardo Becerro-de-Bengoa-Vallejo, Laura Esteban-Gonzalo, Carlos Martin-Villa, César Calvo-Lobo, David Rodriguez-Sanz

**Affiliations:** 1 Facultad de Ciencias de la Salud, Universidad Rey Juan Carlos, 28933 Madrid, Spain; jorgesanidad@gmail.com (J.P.-O.); marta.losa@urjc.es (M.E.L.-I.); 2 Facultad de Enfermería, Fisioterapia y Podología, Universidad Complutense de Madrid, 28040 Madrid, Spain; ribebeva@ucm.es (R.B.-d.-B.-V.); lesteb05@ucm.es (L.E.-G.); podologiamartinvilla@gmail.com (C.M.-V.); cescalvo@ucm.es (C.C.-L.); davidrodriguezsanz@ucm.es (D.R.-S.)

**Keywords:** accuracy, repeatability, inertial

## Abstract

In recent years, interest in finding alternatives for the evaluation of mobility has increased. Inertial measurement units (IMUs) stand out for their portability, size, and low price. The objective of this study was to examine the accuracy and repeatability of a commercially available IMU under controlled conditions in healthy subjects. A total of 36 subjects, including 17 males and 19 females were analyzed with a Wiva Science IMU in a corridor test while walking for 10 m and in a threadmill at 1.6 km/h, 2.4 km/h, 3.2 km/h, 4 km/h, and 4.8 km/h for one minute. We found no difference when we compared the variables at 4 km/h and 4.8 km/h. However, we found greater differences and errors at 1.6 km/h, 2.4 km/h and 3.2 km/h, and the latter one (1.6 km/h) generated more error. The main conclusion is that the Wiva Science IMU is reliable at high speeds but loses reliability at low speeds.

## 1. Introduction

In recent years, there has been increased interest in finding alternatives for the evaluation of mobility, among which inertial measurement units (IMUs) stand out because of their portability, size, and relatively low price [1]. Most publications that include a validation of an IMU compare its performance with optical motion-capture systems [2,3,4,5,6]. So far, the gold standards for gait analysis are optical motion capture systems, force platforms, and plantar pressure platforms, but these systems are expensive, space limited, and time consuming due to the placement of markers on the test subject. IMUs solve all of these problems.

Recent investigations on gait and posture assessment analysis show that an IMU could provide a new perspective for these functional tests as it allows for detailed space, time, and kinematic measurements of human motion on a continuous basis [7]. Mobile motion analysis systems are a promising element in aiding clinical decisions regarding the patient and could provide objective and quantifiable measures of gait, even for physicians with little experience in motion capture [8]. IMUs are increasingly being used for gait analysis because of their validity in healthy patients [9]. IMUs are also being used to study the spatial and temporal parameters of walking as predictors of falls [10] and as predictors of neurological diseases [11].

However, when used as a method of analysis, IMUs must comply with the principles of validity, objectivity, and repeatability [12]. Reliable repeatability is a prerequisite for the evolution of a patient over a period of time. Repeatability is necessary to differentiate between inaccurate measurements and actual changes in a patient’s gait [12]. So far, research shows excellent repeatability for the analysis of gait [13,14], although there are some limitations, such as the calculation of parameters that depend on a spatial relationship of both feet, such as the width or length of the step [15]. Fariboz et al. provided information on the evaluation of the effects of medication in Parkinson’s disease and demonstrated the applicability of inertial sensors to evaluate the disease [16]. The main purpose of this study was to evaluate the accuracy and repeatability of space-time parameters of walking with an IMU, called Wiva science, which is currently marketed, is simple to use and has a relatively low cost. Comparing it to other IMUs also marketed such as The Rehagate system, Physilog GaitUp and APDM Opal, our research team chose this device because it currently has few published studies and we believe that this study will be a novelty and will contribute to improving people’s quality of life.

Therefore, we pose the following question: Are IMUs accurate for assessing gait in healthy patients without gait pathology?

## 2. Methods

In total, 36 healthy subjects participated in the present study. The exclusion criteria were recent and significant ligament damage, surgery, bone fractures, muscle damage in the lower extremities, abnormal gait patterns, contraindications to exercise, or other health conditions that could negatively affect the results of the study. A case-control study was done based on the guidelines of Strengthening the Reporting of Observational Research in Epidemiology (STROBE) [17]. The Declaration of Helsinki and human experimentation rules were followed [18]. This study was approved by the ethics committee of the Universidad Rey Juan Carlos de Madrid (internal registration number 2102201803818), and all participants signed an informed consent form before participating.

**Figure 1:**
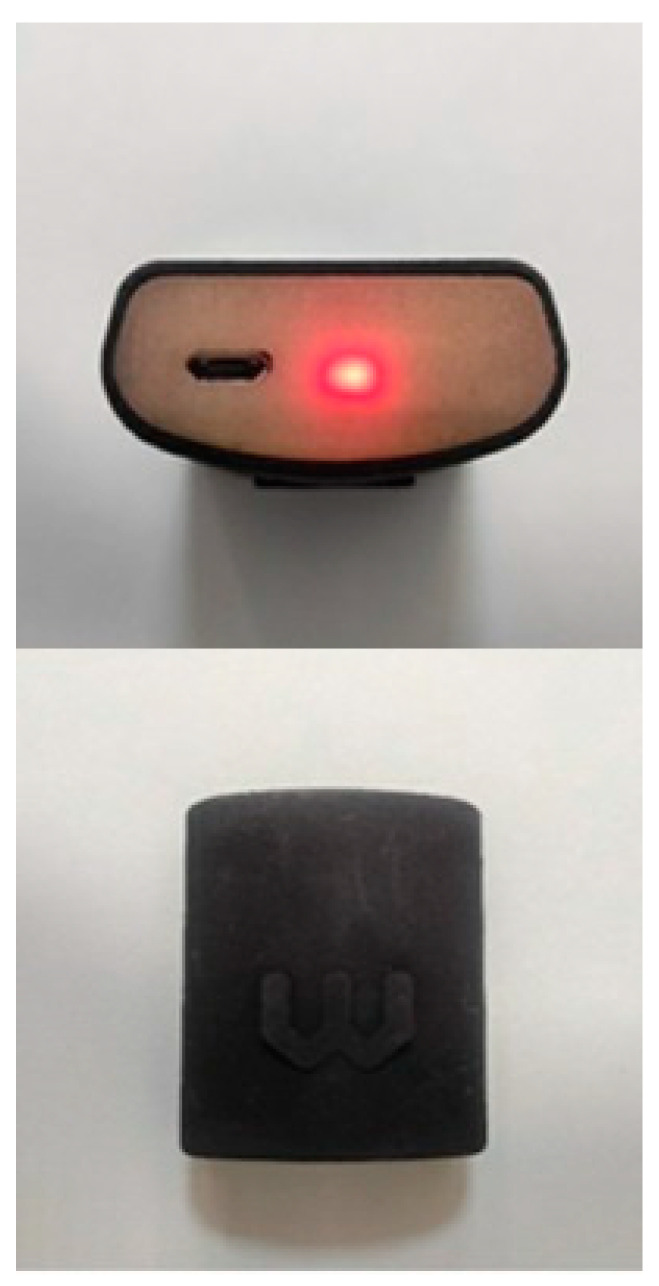
Wiva Science IMU. (Inertial Measurement Unit).

The accuracy and repeatability of a Wiva Science sensor (Wiva Science-LetSense Srl, Bologna, Italy; Figure 1) was tested by a subject walking on a treadmill and on normal ground. The treadmill was used to minimize the variability of walking among people between days [19]. For each condition, the IMU was placed in the sacral area of each subject, which was determined by palpation of the area by the investigator.

Intra-rater reliability (intraclass correlation coefficient (ICC) 0.91–0.98) and inter-rater reliability (ICC 0.80–0.87) have been established [20]. 

The subjects walked on the treadmill at 0.44 m/s (1.6 km/h), 0.67 m/s (2.4 km/h), 0.89 m/s (3.2 km/h), 1.11 m/s (4 km/h), and 1.33 m/s (4.8 km/s) for one minute at each speed. Subjects were given 15 s to adjust to each speed. In order to measure the repeatability of the IMU, the subjects returned for a second test. The protocol and the investigator were the same in both data-collection sessions. Subjects also performed several timed 10 m walking tests while wearing the IMU on normal ground. The subject walked for 10 m in a straight line, which included the subject’s acceleration and deceleration distance. The subjects’ normal speeds were estimated using the Wiva Science system and the 10 m distance. The subjects walked the 10 m three times at their normal speed, and the average was used in the statistical analysis.

The walking parameters of the IMU sensors were extracted using Biomech software (Version 1.6.1.14687, LetSense Group srl., Bologna, Italy, http://letsense.net (accessed on 11 December 2019)). The IMU measurements collected for the tests were validated with the speed data collected from the treadmill. The parameters studied were speed (m/min), step cadence (steps/min), stride length (m), stride length/height (%), average length (Emi) of step 1 (%), average length (Emi) of step 2 (%), average duration of step 1 (%), average duration of step 2 (%), position duration (% walking cycle), oscillation duration (% walking cycle), left foot bearing time (% gait cycle), right foot bearing time (% gait cycle), left foot swing time (% gait cycle), right foot swing time (% gait cycle), and surface speed (10 m). The measurements were taken as clinical standards. All participants did two tests separated by two days. All measurements were recorded by the same researcher.

### 2.1. Sample Size

A heterogeneous study sample was chosen since the measurement instrument is intended for different conditions. With an ICC of 0.90 and a confidence interval of ± 0.1, a sample of 35 participants was considered sufficient to perform the statistical calculations [21]. When testing reliability according to application in individual subjects and for use in clinical practice, a high ICC of 0.9 or 0.95 is recommended to increase the probability of measurement reliability [22,23].

We compared the sample size of this study with other research carried out to date. The RehaGait system was evaluated with 22 healthy subjects at different speeds on a treadmill [24], and Physilog GaitUp was evaluated with 14 individuals with stroke and 25 non-disabled elderly subjects using the “Up and Go” test [25]. The Valedo system was evaluated with 20 healthy subjects [26], the IMU Xsens MTx was evaluated with 10 subjects with Parkinson’s disease in a walking test [27], and the InertiaCube3 was evaluated with 4 participants who had suffered strokes [28]. The IMU Shimmer3 sensor was evaluated with 4 subjects with Parkinson’s disease and 11 healthy subjects [15], and the APDM Opal IMUs was evaluated on a treadmill with 19 healthy subjects and on regular ground with 14 healthy subjects [29].

### 2.2. Statistical Analysis

To interpret the ICC values, we used reference points proposed by Landis and Knoch [30] to indicate the following: 0.20 or less: mild; 0.21–0.40: fair; 0.41–0.60: moderate; 0.61–0.80: substantial; and 0.81 or greater: almost perfect. We followed Portney and Watkins’ guidance that clinical measurements with reliability coefficients greater than 0.90 increase the probability of measurement reliability [23]. For each test within the session and between sessions, the ICC [31,32] was used to evaluate the reliability of each gait parameter.

All data analyses were performed in SPSS for Windows version 22 (SPSS Inc., Chicago, IL, USA). A Kolmogorov-Smirnov test was carried out to assess the normal distribution of the data. A descriptive statistical analysis was performed using the mean ± standard deviation (SD) and the 95% confidence interval. In addition, paired t-tests were performed to evaluate systematic differences in gait parameters between sessions. For the intersession evaluation, the mean value of the 14 measurements was analyzed.

The coefficients of variation (CVs) were calculated for absolute parameter comparison. The CV was calculated to measure the reliability of each session as the mean normalized to the SD. This value represents the variation between the tests normalized to the mean for each variable. A high CV value shows a greater heterogeneity of variable values. The statistical analysis was performed using the data from both feet.

Standard errors of the mean (SEMs) were calculated to measure the range of error for each gear parameter. The SEM was calculated between sessions from the ICC and SD as SEM = $s_{x}.\sqrt{1-r_{xx}}$, where *s_x_* is the SD of the test data set, and *r_xx_*_._ is the confidence coefficient for these data, which is ICC in this case. Finally, the normality values (NVs) of the sample were defined for all the variables obtained with the Wiva Science system (NV = mean ± 1.96 * SD). From the result of each variable, NV was used to calculate the 95% confidence interval. A *p*-value < 0.05 with a 95% confidence interval was considered statistically significant for all tests. 

Moreover, Bland and Altman plots were calculated to check agreement and heteroscedasticity [33].

## 3. Results

There were 36 subjects, including 17 males and 19 females with a mean age of 35.19 ± 11.79 years (19–64 years), mean weight of 74.83 ± 16.91 kg (47–107 kg), mean height of 171.69 ± 7.49 cm (157–192 cm), and mean body mass index (BMI) of 25.23 ± 4.34 (18.4–33.4). We found statistically significant differences in weight, height, and BMI (kg/cm^2^) [34], as shown in Table 1.

High reliability was observed in all measurements in the first session (Table 2) with ICC > 0.81 except for the following variables: the average duration of step 1 at normal speed (ICC 0.41), 1.6, 2.4, 3.2 and 4 km/h (ICC: 0.28, ICC 0.73, ICC 0.39, and ICC 0.73, respectively); the average duration of step 2 at all speeds, which had low reliability (normal, 1.6, 2.4, 3.2 and 4 km/h had ICCs of 0.24, 0.22, 0.73, 0.37, and 0.75, respectively); the variable position duration at 1.6 km/h (ICC 0.72); the variable oscillation duration at 1.6 km/h (ICC 0.77); the variable left foot bearing time at 1.6 km/h and 3.2 km/h (ICC 0.38 and ICC 0.58 respectively); the variable right foot bearing time at normal speed (ICC 0.69) and 1.6 km/h (ICC 0.54); the variable left foot swing time at 1.6 km/h (ICC 0.49); and the variable right foot swing time at 1.6 km/h (ICC 0.48). The SEM was low except at normal speed, 1.6 and 2.4 km/h.

High reliability was observed in all measurements in the second session (Table 3) with ICC > 0.81 except for the following variables: the variable average duration of step 1 at all speeds (1.6 km/h, 2.4 km/h, 3.2 km/h, 4 km/h, 4.8 km/h: ICC 0.51, ICC 0.35, ICC 0.66, ICC 0.67, and ICC 0.31, respectively); the variable average duration of step 2 at all speeds (ICC 0.59, ICC 0.47, ICC 0.50, ICC 0.60, ICC 0.72, and ICC 0.34 respectively); the variable average duration of step 2 4.8 km/h (ICC 0.34); the variable oscillation duration at 1.6 km/h (ICC 0.78); the variable left foot bearing time at 1.6 km/h (ICC 0;63); the variable right foot bearing time at 1.6 km/h and 2.4 km/h (ICC 0.53 and ICC 0.80, respectively); the variable left foot swing time at 1.6 km/h (ICC 0.63); and the variable right foot swing time at 1.6 km/h (ICC 0.56). SEM was low except at normal speed, 1.6 km/h, and 2.4 km/h.

Comparing the differences between first and second session (Table 4), we observed a significant difference between the first and second sessions at normal speed (*p* = 0.05).

## 4. Discussion

Assessing the reliability of any IMU or gait analysis system is essential to ensure the reliability of the measurements made by analyzing the gait parameters, a lack of errors in the operation of the devices, and a lack of human error. This study shows that the Wiva Science IMU has high reliability in its measurements, but we must mention that the reliability is reduced at lower speeds. At 4 km/h, no errors are observed in the measurements, and at 1.6 km/h, the results are most affected. This may be due to changes in the speed of the subject’s walking and errors between both days of the test. 

Furthermore, if we compare our study with others, we agree that ICCs are lower at slower speeds and higher ICCs at higher speeds or with normal gait velocity [24,35].

According to the validation research of the gait parameters studied with the IMU Free4Act [36] which could be classified as the predecessor of Wiva Science, they obtained lower ICC results the lower the study speeds were.

Comparing our investigation with the reliability and repeatability study of the IMU MTw sensors (MTw sensors, Xsens Technologies B.V., The Netherlands) [37] they performed the gait tests at a comfortable speed for the patient, 25 m for 1 min in 19 subjects. They obtained an ICC > 0.8 in all measurements including intrasession and intersession, being in agreement with our study by having good reliability and repeatability at a speed comfortable for the patient.

Furthermore, we believe that since the worst results were isolated and occurred at 1.6, 2.4 and 3.2 km/h speeds, they are not significant. Compared to other studies with high reliability results ICC > 0.81, it has the worst scores in stance time and swing time as our study [15]. With regard to the reproduction of the study, the subjects did not use footwear or clothing that could bias the measurements, such as socks or stockings, since they could alter the biomechanics of the participant.

The results of our research are similar to other researches where they have used inertial measurement units placed on the lower back [38,39] and having greater difficulty in measuring the parameters related to stance and swing times [40,41]. It is possible that this is due to the fact that they are more accurate in their measurements the more proximal their placement is to the foot [42]. However, the placement of the inertial measurement units in the sacral area, according to some authors, can reduce residual errors related to pelvic rotations and errors in gait measurements [43].

All of the research revealed positive results in terms of accuracy and repeatability, so we believe that using a sample of 36 healthy subjects for our research was sufficient. We must emphasize that we first placed the IMU in the subject’s sacral area with an elastic belt supplied by the manufacturer, but it was not useful, and it even skewed the measurements during the repetition of the tests. Therefore, the device was held with hypoallergenic adhesive bandages. Another important limitation was the impossibility of limiting the tests from the Biomech software (Version 1.6.1.14687, LetSense Group srl., Bologna, Italy, http://letsense.net (accessed on 11 December 2019)), which was not able to eliminate the acceleration and deceleration times from the tests performed in a 10 m-long corridor at normal speed. A few studies have quantified events occurring at the initiation of gait using IMUs [44,45]. The total time spent between the two days of the test was approximately one and a half hours per subject, which was a source of fatigue for the participant. 

## 5. Conclusions

We found no difference when we compared the variables between the first and second sessions at higher speeds (4 and 4.8 km/h). However, we found greater differences and errors at lower speeds of 3.2, 2.4 and 1.6 km/h, and the latter one generated more error. Based on the results of this study, we leave open future lines of investigation related to comparison between morphotypes of the foot and the behavior for walking evaluated with IMUs. In addition, we must test Wiva Science IMU at different walking speeds and find whether it is effectively unreliable at lower speeds. We must also evaluate its behavior at higher speeds.

## Figures and Tables

**Table 1:** Demographic data of the sample.

Variable	Men (*n* = 17) Mean ± SD (Range)	Women (*n* = 19) Mean ± SD (Range)	Total (*n* = 36) Mean ± SD (Range)	*p* Value
**AGE (years)**	35.64 ± 13.19 (19–64)	34.78 ± 10.73 (22–62)	35.19 ± 11.79 (19–64)	0.8311
**WEIGHT (kg)**	86.29 ± 13.40 (60–107)	64.57 ± 12.70 (47–90)	74.83 ± 16.91 (47–107)	0.0000 *
**SIZE (cm)**	177.05 ± 5.58 (169–192)	166.89 ± 5.47 (157–177)	171.69 ± 7.49 (157–192)	0.0000 *
**BMI**	27.44 ± 3.55 (20.76–33.4)	23.25 ± 4.08 (18.4–31.14)	25.23 ± 4.34 (18.4–33.4)	0.0025 *

Abbreviations: cm: centimeters; kg: kilograms; BMI: body mass index; SD: standard deviation; 95% CI: 95% confidence interval; *: significant differences, *p* < 0.05.

**Table 2:** Reliability analysis within the variables studied for the first session.

Variables	Mean (DS)	IC95%	CV (%)	ICC (2.1) (IC95%)	SEM	MDC	95% Normality Values
**Normal speed**	73.68 (12.63)	(69.56–77.81)	17.14	0.93	3.18	8.81	(48.93–98.44)
**Speed 1.6 km/h**	34.30 (7.16)	(31.96–36.64)	20.87	0.93	1.77	4.92	(20.27–48.33)
**Speed 2.4 km/h**	43.09 (6.63)	(40.93–45.26)	15.38	0.98	0.99	2.29	(30.10–56.09)
**Speed 3.2 km/h**	54.51 (8.20)	(51.83–57.19)	15.05	0.98	0.99	2.36	(38.42–70.59)
**Speed 4 km/h**	67.39 (9.29)	(64.36–70.43)	13.78	0.98	0.99	3.18	(49.18–85.61)
**Speed 4.8 km/h**	79.27 (10.47)	(75.85–82.69)	13.21	0.98	0.99	3.17	(58.74–99.79)
**Step cadence normal speed**	55.74 (4.29)	(54.34–57.14)	7.70	0.87	1.53	4.26	(47.32–64.16)
**Step cadence 1.6 km/h**	37.04 (7.94)	(34.44–39.64)	21.45	0.93	1.98	5.50	(21.46–52.62)
**Step cadence 2.4 km/h**	42.37 (4.83)	(40.80–43.95)	11.40	0.98	0.59	1.64	(32.90–51.84)
**Step cadence 3.2 km/h**	49.26 (3.99)	(47.96–50.57)	8.11	0.99	0.30	0.83	(41.43–57.10)
**Step cadence 4 km/h**	55.41 (3.41)	(54.30–56.53)	6.17	0.98	0.48	1.34	(48.71–62.12)
**Step cadence 4.8 km/h**	59.76 (3.14)	(58.7 –60.79)	5.26	0.98	0.37	1.02	(53.59–65.94)
**Stride length normal speed**	1.32 (0.25)	(1.25 –1.39)	15.50	0.96	0.04	0.11	(0.92–1.72)
**Stride length 1.6 km/h**	0.94 (0.17)	(0.89–1.00)	18.04	0.92	0.04	0.12	(0.61–1.28)
**Stride length 2.4 km/h**	1.02 (0.15)	(0.97–1.07)	14.95	0.98	0.01	0.05	(0.72–1.32)
**Stride length 3.2 km/h**	1.10 (0.15)	(1.05–1.15)	13.96	0.98	0.01	0.04	(0.80–1.41)
**Stride length 4 km/h**	1.21 (0.16)	(1.16–1.27)	13.48	0.98	0.01	0.05	(0.89–1.54)
**Stride length 4.8 km/h**	1.32 (0.18)	(1.26–1.38)	13.62	0.99	0.01	0.04	(0.97–1.68)
**Stride length/height normal speed**	79.16 (13.31)	(74.81–83.51)	16.81	0.96	2.33	6.46	(53.07–105.26)
**Stride length/height 1.6 km/h**	56.82 (13.30)	(52.48–61.17)	23.41	0.98	1.58	4.38	(30.75–82.90)
**Stride length/height 2.4 km/h**	61.50 (12.13)	(57.53–65.46)	19.72	0.99	1.04	2.90	(37.72–85.27)
**Stride length/height 3.2 km/h**	66.52 (11.91)	(62.63–70.42)	17.91	0.99	1.04	2.90	(43.16–89.89)
**Stride length/height 4 km/h**	73.07 (12.37)	(69.02–77.11)	16.93	0.99	1.09	3.02	(48.81–97.32)
**Stride length/height 4.8 km/h**	79.72 (13.76)	(75.22–84.22)	17.27	0.99	1.02	2.82	(52.73–106.71)
**Average length of step 1—normal speed**	0.66 (0.10)	(0.62–0.69)	15.98	0.95	0′02	0.06	(0.45–0.86)
**Average length of step 1—1.6 km/h**	0.47 (0.08)	(0.44–0.49)	17.64	0.95	0.01	0.04	(0.30–0.63)
**Average length of step 1—2.4 km/h**	0.51 (0.08)	(0.48–0.54)	16.07	0.98	0.01	0.03	(0.35–0.67)
**Average length of step 1—3.2 km/h**	0.55 (0.08)	(0.52–0.57)	15.16	0.98	0.01	0.03	(0.38–0.71)
**Average length of step 1—4 km/h**	0.60 (0.08)	(0.58–0.63)	13.95	0.97	0.01	0.03	(0.44–0.77)
**Average length of step 1—4.8 km/h**	0.66 (0.09)	(0.63–0.69)	14.20	0.98	0.01	0.03	(0.48–0.85)
**Average length of step 2—normal speed**	0.66 (0.10)	(0.62–0.69)	15.89	0.94	0.02	0.06	(0.45–0.86)
**Average length of step 2—1.6 km/h**	0.47 (0.08)	(0.44–0.49)	17.12	0.95	0.01	0.04	(0.31–0.62)
**Average length of step 2—2.4 km/h**	0.50 (0.07)	(0.48–0.53)	15.16	0.97	0.01	0.03	(0.35–0.65)
**Average length of step 2—3.2 km/h**	0.55 (0.07)	(0.53–0.58)	13.80	0.98	0.01	0.03	(0.40–0.70)
**Average length of step 2—4 km/h**	0.61 (0.08)	(0.58–0.63)	13.78	0.97	0.01	0.03	(0.44–0.77)
**Average length of step 2—4.8 km/h**	0.66 (0.09)	(0.63–0.69)	13.57	0.98	0.01	0.02	(0.48–0.83)
**Average duration of step 1—normal speed**	49.89 (2.18)	(49.17–50.60)	4.37	0.41	1.66	4.62	(45.61–54.17)
**Average duration of step 1—1.6 km/h**	49.42 (2.36)	(48.65–50.20)	4.77	0.28	2.00	5.55	(44.79–54.05)
**Average duration of step 1—2.4 km/h**	50.09 (1.58)	(49.57–50.69)	3.16	0.73	0.82	2.28	(46.98–53.20)
**Average duration of step 1—3.2 km/h**	49.82 (1.14)	(49.45–50.19)	2.29	0.39	0.89	2.46	(47.58–52.06)
**Average duration of step 1—4 km/h**	49.79 (1.17)	(49.40–50.17)	2.35	0.73	0.60	1.68	(47.49–52.08)
**Average duration of step 1—4.8 km/h**	49.97 (0.91)	(49.67–50.27)	1.83	0.88	0.31	0.87	(48.17–51.76)
**Average duration of step 2—normal speed**	50.29 (2.53)	(49.46–51.12)	5.04	0.24	2.20	6.12	(45.31–55.27)
**Average duration of step 2—1.6 km/h**	50.63 (2.28)	(49.88–51.38)	4.51	0.22	2.01	5.58	(46.15–55.11)
**Average duration of step 2—2.4 km/h**	49.92 (1.60)	(49.39–50.44)	3.21	0.73	0.82	2.27	(46.77–53.06)
**Average duration of step 2—3.2 km/h**	50.18 (1.14)	(49.80–50.55)	2.28	0.37	0.90	2.50	(47.93–52.42)
**Average duration of step 2—4 km/h**	50.21 (1.17)	(49.83–50.62)	2.33	0.75	0.58	1.62	(47.92–52.51)
**Average duration of step 2—4.8 km/h**	50.03 (0.91)	(49.73–50.33)	1.83	0.88	0.31	0.88	(48.23–51.83)
**Position duration normal speed**	63.67 (2.63)	(62.81–64.53)	4.14	0.91	0.75	2.10	(58.50–68.84)
**Position duration 1.6 km/h**	62.39 (2.56)	(61.55–63.22)	4.11	0.72	1.34	3.72	(57.35–67.42)
**Position duration 2.4 km/h**	62.92 (2.31)	(62.16–63.67)	3.67	0.88	0.77	2.13	(58.39–67.45)
**Position duration 3.2 km/h**	61.80 (1.99)	(61.14–62.45)	3.22	0.94	0.46	1.29	(57.88–65.71)
**Position duration 4 km/h**	60.98 (2.01)	(60.32–61.64)	3.30	0.98	0.28	0.78	(57.03–64.94)
**Position duration 4.8 km/h**	60.09 (1.83)	(59.48–60.69)	3.05	0.98	0.23	0.64	(56.48–63.69)
**Oscillation duration normal speed**	34.34 (2.72)	(33.45–35.23)	7.93	0.93	0.67	1.85	(29.00–39.68)
**Oscillation duration 1.6 km/h**	36.46 (2.41)	(35.67–37.25)	6.61	0.77	1.14	3.16	(31.74–41.19)
**Oscillation duration 2.4 km/h**	35.65 (2.33)	(34.89–36.41)	6.53	0.88	0.78	2.17	(31.08–40.22)
**Oscillation duration 3.2 km/h**	36.55 (2.03)	(35.89–37.21)	5.55	0.94	0.46	1.27	(32.57–40.53)
**Oscillation duration 4 km/h**	37.14 (2.02)	(36.48–37.80)	5.43	0.98	0.27	0.76	(33.18–41.10)
**Oscillation duration 4.8 km/h**	37.91 (1.80)	(37.32–38.50)	4.76	0.98	0.23	0.64	(34.37–41.45)
**Left foot bearing time normal speed**	63.62 (3.48)	(62.48–64.76)	5.47	0.91	0.99	2.74	(56.79–70.45)
**Left foot bearing time 1.6 km/h**	61.88 (3.68)	(60.67–63.08)	5.95	0.38	2.89	8.01	(54.65–69.10)
**Left foot bearing time 2.4 km/h**	62.93 (2.43)	(62.13–63.72)	3.82	0.89	0.80	2.23	(58.16–67.69)
**Left foot bearing time 3.2 km/h**	62.12 (3.62)	(60.94–63.30)	5.83	0.58	2.32	6.45	(55.02–69.22)
**Left foot bearing time 4 km/h**	60.85 (2.42)	(60.06–61.64)	3.97	0.94	0.57	1.59	(56.11–65.60)
**Left foot bearing time 4.8 km/h**	59.84 (2.23)	(59.11–60.57)	3.73	0.94	0.50	1.40	(55.46–64.22)
**Right foot bearing time normal speed**	63.72 (2.75)	(62.82–64.62)	4.32	0.69	1.53	4.24	(58.32–69.11)
**Right foot bearing time 1.6 km/h**	62.90 (3.43)	(61.78–64.03)	5.45	0.54	2.31	6.41	(56.18–69.63)
**Right foot bearing time 2.4 km/h**	62.91 (2.89)	(61.96–63.85)	4.59	0.83	1.17	3.24	(57.24–68.57)
**Right foot bearing time 3.2 km/h**	61.75 (2.10)	(61.06–62.44)	3.41	0.86	0.77	2.14	(57.62–65.88)
**Right foot bearing time 4 km/h**	61.12 (2.27)	(60.38–61.87)	3.71	0.92	0.62	1.74	(56.67–65.58)
**Right foot bearing time 4.8 km/h**	60.34 (2.02)	(59.67–61.00)	3.35	0.91	0.59	1.65	(56.36–64.31)
**Left foot swing time normal speed**	34.37 (3.54)	(33.21–35.53)	10.31	0.94	0.80	2.24	(27.42–41.32)
**Left foot swing time 1.6 km/h**	37.02 (3.33)	(35.93–38.11)	9.00	0.49	2.37	6.57	(30.48–43.56)
**Left foot swing time 2.4 km/h**	35.65 (2.43)	(34.85–36.45)	6.83	0.89	0.80	2.23	(30.87–40.43)
**Left foot swing time 3.2 km/h**	36.51 (2.40)	(35.72–37.29)	6.58	0.93	0.59	1.66	(31.80–41.22)
**Left foot swing time 4 km/h**	37.29 (2.42)	(36.49–38.08)	6.49	0.94	0.57	1.59	(32.54–42.03)
**Left foot swing time 4.8 km/h**	38.16 (2.20)	(37.44–38.87)	5.76	0.94	0.50	1.40	(33.84–42.47)
**Right foot swing time normal speed**	34.18 (2.71)	(33.30–35.07)	7.94	0.90	0.83	2.32	(28.86–39.51)
**Right foot swing time 1.6 km/h**	35.81 (3.60)	(34.64–36.99)	10.06	0.48	2.58	7.15	(28.75–42.88)
**Right foot swing time 2.4 km/h**	35.65 (2.92)	(34.69–36.60)	8.19	0.83	1.19	3.31	(29.92–41.37)
**Right foot swing time 3.2 km/h**	36.59 (2.14)	(35.89–37.29)	5.86	0.87	0.76	2.11	(32.39–40.80)
**Right foot swing time 4 km/h**	37.01 (2.27)	(36.27–37.75)	6.14	0.92	0.62	1.72	(32.56–41.47)
**Right foot swing time 4.8 km/h**	37.62 (2.01)	(37.01–38.33)	5.35	0.91	0.59	1.65	(33.71–41.62)

Abbreviations: km/h (kilometers per hour); IC (confidence interval); CV (coefficient of variation); ICC (coefficient of intraclass correlation); SEM (standard error of mean); MDC (minimum detectable change).

**Table 3:** Reliability analysis within the variables studied for the second session.

Variables	MEAN (DS)	IC95%	CV (%)	ICC (2,1) (IC95%)	SEM	MDC	95% NORMALITY VALUES
**NORMAL SPEED**	76.21 (11.86)	(72.34–80.08)	15.56	0.96	2.32	6.44	(52.96–99.45)
**SPEED 1.6 km/h**	34.77 (6.50)	(32.64–36.89)	18.70	0.93	1.71	4.75	(22.01–47.52)
**SPEED 2.4 km/h**	43.11 (6.52)	(40.98–45.24)	15.12	0.93	1.66	4.60	(30.33–55.89)
**SPEED 3.2 km/h**	55.03 (7.07)	(52.72–57.34)	12.85	0.98	0.74	2.06	(41.16–68.90)
**SPEED 4 km/h**	67.61 (9.14)	(64.62–70.59)	13.52	0.99	0.90	2.50	(49.68–85.53)
**SPEED 4.8 km/h**	79.79 (10.79)	(76.26–83.31)	13.52	0.98	1.15	3.18	(58.63–100.95)
**STEP CADENCE NORMAL SPEED**	56.90 (3.80)	(55.66–58.14)	6.68	0.96	0.67	1.86	(49.45–64.35)
**STEP CADENCE 1.6 km/h**	34.73 (6.15)	(32.72–36.74)	17.72	0.90	1.89	5.25	(22.66–46.80)
**STEP CADENCE 2.4 km/h**	40.74 (5.31)	(39.00–42.48)	13.04	0.88	1.82	5.06	(30.32–51.16)
**STEP CADENCE 3.2 km/h**	48.62 (3.63)	(47.43–49.81)	7.47	0.98	0.42	1.16	(41.50–55.74)
**STEP CADENCE 4 km/h**	55.13 (3.29)	(54.05–56.21)	5.97	0.99	0.25	0.70	(48.67–61.59)
**STEP CADENCE 4.8 km/h**	59.57 (3.07)	(58.57–60.58)	5.16	0.96	0.54	1.50	(53.55–65.60)
**STRIDE LENGTH NORMAL SPEED**	1.34 (0.19)	(1.27–1.40)	14.64	0.97	0.03	0.09	(0.95–1.72)
**STRIDE LENGTH 1.6 km/h**	1.01 (0.16)	(0.95–1.06)	16.42	0.98	0.02	0.05	(0.68–1.33)
**STRIDE LENGTH 2.4 km/h**	1.058 (0.13)	(1.01–1.10)	12.97	0.98	0.01	0.04	(0.78–1.32)
**STRIDE LENGTH 3.2 km/h**	1.13 (0.14)	(1.08–1.18)	12.55	0.99	0.01	0.03	(0.85–1.41)
**STRIDE LENGTH 4 km/h**	1.23 (0.17)	(1.17–0.28)	13.80	0.99	0.01	0.04	(0.89–1.56)
**STRIDE LENGTH 4.8 km/h**	1.34 (0.18)	(1.28–1.40)	14.02	0.99	0.01	0.04	(0.97–1.71)
**STRIDE LENGTH/HEIGHT NORMAL SPEED**	80.32 (12.34)	(76.29–84.35)	15.36	0.97	1.86	5.16	(56.13–104.51)
**STRIDE LENGTH/HEIGHT 1.6 km/h**	60.79 (12.73)	(56.64–64.95)	20.93	0.99	1.16	3.24	(35.84–85.75)
**STRIDE LENGTH/HEIGHT 2.4 km/h**	63.51 (11.29)	(59.82–67.20)	17.78	0.99	0.87	2.43	(41.37–85.65)
**STRIDE LENGTH/HEIGHT 3.2 km/h**	68.02 (10.93)	(64.45–71.59)	16.07	0.99	0.79	2.19	(46.59–89.45)
**STRIDE LENGTH/HEIGHT 4 km/h**	73.67 (12.37)	(69.63–77.71)	16.79	0.99	0.87	2.43	(49.43–97.92)
**STRIDE LENGTH/HEIGHT 4.8 km/h**	80.50 (13.92)	(75.95–85.04)	17.29	0.99	0.85	2.37	(53.20–107.79)
**AVERAGE LENGTH OF STEP 1 NORMAL SPEED**	0.67 (0.10)	(0.63–0.70)	15.00	0.96	0.02	0.05	(0.47–0.86)
**AVERAGE LENGTH OF STEP 1 1.6 km/h**	0.51 (0.08)	(0.48–0.53)	17.09	0.97	0.01	0.04	(0.33–0.68)
**AVERAGE LENGTH OF STEP 1 2.4 km/h**	0.53 (0.07)	(0.50–0.55)	13.73	0.96	0.01	0.04	(0.38–0.67)
**AVERAGE LENGTH OF STEP 1 3.2 km/h**	0.56 (0.07)	(0.53–0.58)	13.46	0.98	0.01	0.02	(0.41–0.71)
**AVERAGE LENGTH OF STEP 1 4 km/h**	0.61 (0.08)	(0.58–0.64)	14.08	0.98	0.01	0.02	(0.44–0.78)
**AVERAGE LENGTH OF STEP 1 4.8 km/h**	0.67 (0.09)	(0.64–0.70)	14.35	0.99	0.01	0.02	(0.48–0.86)
**AVERAGE LENGTH OF STEP 2 NORMAL SPEED**	0.67 (0.10)	(0.63–0.70)	15.12	0.96	0.02	0.05	(0.47–0.87)
**AVERAGE LENGTH OF STEP 2 1.6 km/h**	0.50 (0.08)	(0.47–0.52)	17.07	0.97	0.01	0.03	(0.33–0.66)
**AVERAGE LENGTH OF STEP 2 2.4 km/h**	0.52 (0.07)	(0.50–0.54)	13.68	0.96	0.01	0.03	(0.38–0.66)
**AVERAGE LENGTH OF STEP 2 3.2 km/h**	0.57 (0.07)	(0.54–0.59)	12.51	0.97	0.01	0.02	(0.43–0.70)
**AVERAGE LENGTH OF STEP 2 4 km/h**	0.61 (0.08)	(0.58–0.64)	14.39	0.98	0.01	0.02	(0.44–0.78)
**AVERAGE LENGTH OF STEP 2 4.8 km/h**	0.66 (0.09)	(0.63–0.70)	14.32	0.98	0.01	0.02	(0.48–0.85)
**AVERAGE DURATION OF STEP 1 NORMAL SPEED**	49.93 (2.36)	(49.16–50.71)	4.74	0.51	1.65	4.57	(45.29–54.58)
**AVERAGE DURATION OF STEP 1 1.6 km/h**	50.05 (2.98)	(49.08–51.03)	5.96	0.35	2.39	6.64	(44.20–55.91)
**AVERAGE DURATION OF STEP 1 2.4 km/h**	49.75 (1.67)	(49.20–50.30)	3.37	0.66	0.97	2.69	(46.47–53.04)
**AVERAGE DURATION OF STEP 1 3.2 km/h**	49.98 (1.31)	(49.55–50.41)	2.63	0.60	0.82	2.29	(47.39–52.56)
**AVERAGE DURATION OF STEP 1 4 km/h**	49.84 (1.07)	(49.48–50.19)	2.15	0.67	0.61	1.69	(47.73–51.94)
**AVERAGE DURATION OF STEP 1 4.8 km/h**	50.05 (1.12)	(49.68–50.41)	2.23	0.31	0.92	2.56	(47.85–52.24)
**AVERAGE DURATION OF STEP 2 NORMAL SPEED**	50.31 (2.09)	(49.62–50.99)	4.16	0.59	1.33	3.69	(46.20–54.42)
**AVERAGE DURATION OF STEP 2 1.6 km/h**	49.78 (3.14)	(48.75–50.80)	6.31	0.47	2.28	6.33	(43.61–55.94)
**AVERAGE DURATION OF STEP 2 2.4 km/h**	50.08 (2.01)	(49.43–50.74)	4.02	0.50	1.41	3.92	(46.13–54.03)
**AVERAGE DURATION OF STEP 2 3.2 km/h**	50.01 (1.30)	(49.58–50.44)	2.61	0.60	0.82	2.28	(47.44–52.57)
**AVERAGE DURATION OF STEP 2 4 km/h**	50.15 (1.07)	(49.80–50.50)	2.14	0.72	0.56	1.57	(48.04–52.25)
**AVERAGE DURATION OF STEP 2 4.8 km/h**	49.95 (1.11)	(49.59–50.31)	2.22	0.34	0.90	2.49	(47.77–52.13)
**POSITION DURATION NORMAL SPEED**	63.63 (2.86)	(62.69–64.56)	4.50	0.93	0.72	2.02	(58.00–69.25)
**POSITION DURATION 1.6 km/h**	63.25 (3.04)	(62.26–64.25)	4.81	0.82	1.28	3.56	(57.28–69.23)
**POSITION DURATION 2.4 km/h**	63.09 (2.78)	(62.19–64.00)	4.41	0.90	0.85	2.35	(57.64–68.55)
**POSITION DURATION 3.2 km/h**	61.77 (2.02)	(61.05–62.48)	3.56	0.98	0.30	0.84	(57.45–66.08)
**POSITION DURATION 4 km/h**	61.03 (2.28)	(60.29–61.78)	3.73	0.98	0.26	0.72	(56.56–65.50)
**POSITION DURATION 4.8 km/h**	60.13 (1.88)	(59.51–60.74)	3.13	0.98	0.24	0.68	(56.44–63.82)
**OSCILLATION DURATION NORMAL SPEED**	34.28 (2.72)	(33.39–35.17)	7.96	0.95	0.56	1.55	(28.93–39.63)
**OSCILLATION DURATION 1.6 km/h**	35.57 (3.12)	(34.55–36.59)	8.77	0.78	1.44	4.00	(29.45–41.69)
**OSCILLATION DURATION 2.4 km/h**	35.48 (2.72)	(34.59–36.37)	7.67	0.92	0.75	2.08	(30.14–40.82)
**OSCILLATION DURATION 3.2 km/h**	36.61 (2.24)	(35.88–37.34)	6.11	0.98	0.30	0.85	(32.22–41.00)
**OSCILLATION DURATION 4 km/h**	37.08 (2.29)	(36.33–37.83)	6.19	0.97	0.35	0.99	(32.58–41.58)
**OSCILLATION DURATION 4.8 km/h**	37.84 (1.89)	(37.22–38.46)	5.00	0.97	0.31	0.85	(34.13–41.56)
**LEFT FOOT BEARING TIME NORMAL SPEED**	63.45 (3.58)	(62.28–64.63)	5.65	0.92	0.99	2.75	(56.42–70.49)
**LEFT FOOT BEARING TIME 1.6 km/h**	63.53 (4.41)	(62.09–64.97)	6.94	0.63	2.65	7.35	(54.88–72.18)
**LEFT FOOT BEARING TIME 2.4 km/h**	63.06 (2.79)	(62.14–63.97)	4.42	0.87	0.98	2.73	(57.59–68.52)
**LEFT FOOT BEARING TIME 3.2 km/h**	61.99 (2.43)	(61.20–62.79)	3.92	0.96	0.45	1.24	(57.22–66.76)
**LEFT FOOT BEARING TIME 4 km/h**	60.87 (2.60)	(60.02–61.72)	4.27	0.95	0.52	1.46	(55.77–65.96)
**LEFT FOOT BEARING TIME 4.8 km/h**	59.98 (2.40)	(59.20–60.77)	4.00	0.91	0.69	1.92	(55.27–64.69)
**RIGHT FOOT BEARING TIME NORMAL SPEED**	63.80 (2.64)	(62.93–64.66)	4.15	0.83	1.06	2.95	(58.60–68.99)
**RIGHT FOOT BEARING TIME 1.6 km/h**	62.99 (4.50)	(61.51–64.43)	7.15	0.53	3.07	8.52	(54.15–71.82)
**RIGHT FOOT BEARING TIME 2.4 km/h**	63.08 (3.56)	(61.92–64.25)	5.65	0.80	1.58	4.39	(56.09–70.08)
**RIGHT FOOT BEARING TIME 3.2 km/h**	61.58 (2.65)	(60.71–62.44)	4.30	0.94	0.62	1.73	(56.38–66.77)
**RIGHT FOOT BEARING TIME 4 km/h**	61.20 (2.48)	(60.39–62.01)	4.06	0.97	0.40	1.12	(56.32–66.07)
**RIGHT FOOT BEARING TIME 4.8 km/h**	60.27 (1.96)	(59.63–60.91)	3.25	0.91	0.59	1.63	(56.42–64.12)
**LEFT FOOT SWING TIME NORMAL SPEED**	34.54 (3.42)	(33.33–35.57)	9.94	0.95	0.72	2.01	(27.73–41.16)
**LEFT FOOT SWING TIME 1.6 km/h**	35.29 (4.24)	(33.90–36.62)	12.02	0.63	2.57	7.14	(26.97–43.61)
**LEFT FOOT SWING TIME 2.4 km/h**	35.46 (2.91)	(34.51–36.41)	8.21	0.83	1.19	3.30	(29.75–41.16)
**LEFT FOOT SWING TIME 3.2 km/h**	36.38 (2.44)	(35.58–37.18)	6.72	0.96	0.45	1.27	(31.58–41.17)
**LEFT FOOT SWING TIME 4 km/h**	37.28 (2.59)	(36.43–38.12)	6.95	0.95	0.53	1.47	(32.19–42.36)
**LEFT FOOT SWING TIME 4.8 km/h**	38.03 (2.37)	(37.25–38.80)	6.25	0.91	0.69	1.91	(33.37–42.68)
**RIGHT FOOT SWING TIME NORMAL SPEED**	34.13 (2.60)	(33.28–34.98)	7.64	0.84	1.01	2.80	(29.02–39.24)
**RIGHT FOOT SWING TIME 1.6 km/h**	35.83 (4.70)	(34.30–37.37)	13.13	0.56	3.11	8.62	(26.61–45.06)
**RIGHT FOOT SWING TIME 2.4 km/h**	35.57 (3.38)	(34.46–36.68)	9.52	0.84	1.31	3.64	(28.93–42.21)
**RIGHT FOOT SWING TIME 3.2 km/h**	36.84 (2.63)	(35.98–37.70)	7.15	0.95	0.56	1.55	(31.67–42.00)
**RIGHT FOOT SWING TIME 4 km/h**	36.95 (2.48)	(36.13–37.76)	6.73	0.97	0.42	1.16	(32.07–41.82)
**RIGHT FOOT SWING TIME 4.8 km/h**	37.72 (1.94)	(37.09–38.36)	5.16	0.90	0.59	1.63	(33.90–41.54)

Abbreviations: km/h (kilometers per hour); IC (confidence interval); CV (coefficient of Variation); ICC (intraclass correlation coefficient); SEM (standard error of mean); MDC (minimum detectable change).

**Table 4:** Systematic differences between the first and second session.

Variables	Mean (DS) First Session	IC95%	Mean (DS) Second Session	IC95%	LoA	*p* VALUE
**Normal speed**	73.68 (12.63)	(69.56–77.81)	76.21 (11.86)	(72.34–80.08)	−2.52 (−12.48–7.43)	0.005 *
**Speed 1.6 km/h**	34.30 (7.16)	(31.96–36.64)	34.77 (6.50)	(32.64–36.89)	−0.47 (−10.39–9.46)	0.585
**Speed 2.4 km/h**	43.09 (6.63)	(40.93–45.26)	43.11 (6.52)	(40.98–45.24)	−0.02 (−5.15–5.12)	0.969
**Speed 3.2 km/h**	54.51 (8.20)	(51.83–57.19)	55.03 (7.07)	(52.72–57.34)	−0.52 (−6.19–5.15)	0.287
**Speed 4 km/h**	67.39 (9.29)	(64.36–70.43)	67.61 (9.14)	(64.62–70.59)	−0.21 (−4.43–4.01)	0.556
**Speed 4.8 km/h**	79.27 (10.47)	(75.85–82.69)	79.79 (10.79)	(76.26–83.31)	−0.52 (−4.72–3.68)	0.154
**Step cadence normal speed**	55.74 (4.29)	(54.34–57.14)	56.90 (3.80)	(55.66–58.14)	−1.16 (−4.49–2.17)	0.000 *
**Step cadence 1.6 km/h**	37.04 (7.94)	(34.44–39.64)	34.73 (6.15)	(32.72–36.74)	2.31 (−7.53–12.16)	0.009
**Step cadence 2.4 km/h**	42.37 (4.83)	(40.80–43.95)	40.74 (5.31)	(39.00–42.48)	1.63 (−3.01–6.27)	0.000 *
**Step cadence 3.2 km/h**	49.26 (3.99)	(47.96–50.57)	48.62 (3.63)	(47.43–49.81)	0.64 (−2.14–3.43)	0.010 *
**Step cadence 4 km/h**	55.41 (3.41)	(54.30–56.53)	55.13 (3.29)	(54.05–56.21)	0.28 (−1.69–2.26)	0.101
**Step cadence 4.8 km/h**	59.76 (3.14)	(58.73–60.79)	59.57 (3.07)	(58.57–60.58)	0.19 (−2.23–2.61)	0.368
**Stride length normal speed**	1.32 (0.25)	(1.25–1.39)	1.34 (0.19)	(1.27–1.40)	−0.02 (−0.14–0.10)	0.069
**Stride length 1.6 km/h**	0.94 (0.17)	(0.89–1.00)	1.01 (0.16)	(0.95–1.06)	−0.06 (−0.24–0.11)	0.000 *
**Stride length 2.4 km/h**	1.02 (0.15)	(0.97–1.07)	1.058 (0.13)	(1.01–1.10)	−0.03 (−0.16–0.09)	0.005 *
**Stride length 3.2 km/h**	1.10 (0.15)	(1.05–1.15)	1.13 (0.14)	(1.08–1.18)	−0.03 (−0.16–0.11)	0.032 *
**Stride length 4 km/h**	1.21 (0.16)	(1.16–1.27)	1.23 (0.17)	(1.17–1.28)	−0.01 (−0.09–0.07)	0.117
**Stride length 4.8 km/h**	1.32 (0.18)	(1.26–1.38)	1.34 (0.18)	(1.28–1.40)	−0.01 (−0.09–0.06)	0.056
**Stride length/height normal speed**	79.16 (13.31)	(74.81–83.51)	80.32 (12.34)	(76.29–84.35)	−1.16 (−8.69–6.37)	0.079
**Stride length/height 1.6 km/h**	56.82 (13.30)	(52.48–61.17)	60.79 (12.73)	(56.64–64.95)	−3.97 (−14.21–6.27)	0.000 *
**Stride length/height 2.4 km/h**	61.50 (12.13)	(57.53–65.46)	63.51 (11.29)	(59.82–67.20)	−2.01 (−9.86–5.84)	0.005 *
**Stride length/height 3.2 km/h**	66.52 (11.91)	(62.63–70.42)	68.02 (10.93)	(64.45–71.59)	−1.50 (−9.60–6.60)	0.036 *
**Stride length/height 4 km/h**	73.07 (12.37)	(69.02–77.11)	73.67 (12.37)	(69.63–77.71)	−0.61 (−5.37–4.15)	0.143
**Stride length/height 4.8 km/h**	79.72 (13.76)	(75.22–84.22)	80.50 (13.92)	(75.95–85.04)	−0.78 (−5.45–3.90)	0.060
**Average length step 1 normal speed**	0.66 (0.10)	(0.62–0.69)	0.67 (0.10)	(0.63–0.70)	−0.01 (−0.07–0.05)	0.058
**Average length step 1 1.6 km/h**	0.47 (0.08)	(0.44–0.49)	0.51 (0.08)	(0.48–0.53)	−0.04 (−0.12–0.04)	0.000 *
**Average length step 1 2.4 km/h**	0.51 (0.08)	(0.48–0.54)	0.53 (0.07)	(0.50–0.55)	−0.02 (−0.08–0.04)	0.004 *
**Average length step 1 3.2 km/h**	0.55 (0.08)	(0.52–0.57)	0.56 (0.07)	(0.53–0.58)	−0.01 (−0.08–0.05)	0.026*
**Average length step 1 4 km/h**	0.60 (0.08)	(0.58–0.63)	0.61 (0.08)	(0.58–0.64)	−0.01 (−0.05–0.04)	0.140
**Average length step 1 4.8 km/h**	0.66 (0.09)	(0.63–0.69)	0.67 (0.09)	(0.64–0.70)	−0.01 (−0.05–0.04)	0.103
**Average length step 2 normal speed**	0.66 (0.10)	(0.62–0.69)	0.67 (0.10)	(0.63–0.70)	−0.01 (−0.08–0.06)	0.156
**Average length step 2 1.6 km/h**	0.47 (0.08)	(0.44–0.49)	0.50 (0.08)	(0.47–0.52)	−0.03 (−0.12–0.06)	0.001 *
**Average length step 2 2.4 km/h**	0.50 (0.07)	(0.48–0.53)	0.52 (0.07)	(0.50–0.54)	−0.02 (−0.09–0.06)	0.019 *
**Average length step 2 3.2 km/h**	0.55 (0.07)	(0.53–0.58)	0.57 (0.07)	(0.54–0.59)	−0.01 (−0.08–0.05)	0.061
**Average length step 2 4 km/h**	0.61 (0.08)	(0.58–0.63)	0.61 (0.08)	(0.58–0.64)	0.00 (−0.04–0.03)	0.202
**Average length step 2 4.8 km/h**	0.66 (0.09)	(0.63–0.69)	0.66 (0.09)	(0.63–0.70)	−0.01 (−0.05–0.03)	0.070
**Average duration of step 1 normal speed**	49.89 (2.18)	(49.17–50.60)	49.93 (2.36)	(49.16–50.71)	−0.05 (−2.44–2.34)	0.811
**Average duration of step 1 1.6 km/h**	49.42 (2.36)	(48.65–50.20)	50.05 (2.98)	(49.08–51.03)	−0.63 (−4.69–3.43)	0.078
**Average duration of step 1 2.4 km/h**	50.09 (1.58)	(49.57–50.69)	49.75 (1.67)	(49.20–50.30)	0.34 (−1.81–2.48)	0.075
**Average duration of step 1 3.2 km/h**	49.82 (1.14)	(49.45–50.19)	49.98 (1.31)	(49.55–50.41)	−0.16 (−1.61–1.29)	0.208
**Average duration of step 1 4 km/h**	49.79 (1.17)	(49.40–50.17)	49.84 (1.07)	(49.48–50.19)	−0.05 (−1.81–1.71)	0.739
**Average duration of step 1 4.8 km/h**	49.97 (0.91)	(49.67–50.27)	50.05 (1.12)	(49.68–50.41)	−0.08 (−1.41–1.25)	0.481
**Average duration of step 2 normal speed**	50.29 (2.53)	(49.46–51.12)	50.31 (2.09)	(49.62–50.99)	−0.02 (−2.45–2.42)	0.932
**Average duration of step 2 1.6 km/h**	50.63 (2.28)	(49.88–51.38)	49.78 (3.14)	(48.75–50.80)	0.85 (−3.69–5.39)	0.034 *
**Average duration of step 2 2.4 km/h**	49.92 (1.60)	(49.39–50.44)	50.08 (2.01)	(49.43–50.74)	−0.17 (−2.54–2.21)	0.415
**Average duration of step 2 3.2 km/h**	50.18 (1.14)	(49.80–50.55)	50.01 (1.30)	(49.58–50.44)	0.17 (−1.22–1.55)	0.165
**Average duration of step 2 4 km/h**	50.21 (1.17)	(49.83–50.62)	50.15 (1.07)	(49.80–50.50)	0.07 (−1.83–1.96)	0.680
**Average duration of step 2 4.8 km/h**	50.03 (0.91)	(49.73–50.33)	49.95 (1.11)	(49.59–50.31)	0.08 (−1.26–1.42)	0.507
**Position duration normal speed**	63.67 (2.63)	(62.81–64.53)	63.63 (2.86)	(62.69–64.56)	0.04 (−1.79–1.88)	0.780
**Position duration 1.6 km/h**	62.39 (2.56)	(61.55–63.22)	63.25 (3.04)	(62.26–64.25)	−0.87 (−4.63–2.90)	0.010 *
**Position duration 2.4 km/h**	62.92 (2.31)	(62.16–63.67)	63.09 (2.78)	(62.19–64.00)	−0.18 (−3.03–2.67)	0.467
**Position duration 3.2 km/h**	61.80 (1.99)	(61.14–62.45)	61.77 (2.02)	(61.05–62.48)	0.03 (−1.82–1.88)	0.852
**Position duration 4 km/h**	60.98 (2.01)	(60.32–61.64)	61.03 (2.28)	(60.29–61.78)	−0.05 (−1.59–1.49)	0.695
**Position duration 4.8 km/h**	60.09 (1.83)	(59.48–60.69)	60.13 (1.88)	(59.51–60.74)	−0.04 (−1.39–1.30)	0.723
**Oscillation duration normal speed**	34.34 (2.72)	(33.45–35.23)	34.28 (2.72)	(33.39–35.17)	0.06 (−1.68–1.80)	0.684
**Oscillation duration 1.6 km/h**	36.46 (2.41)	(35.67–37.25)	35.57 (3.12)	(34.55–36.59)	0.89 (−2.29–4.08)	0.002 *
**Oscillation duration 2.4 km/h**	35.65 (2.33)	(34.89–36.41)	35.48 (2.72)	(34.59–36.37)	0.17 (−2.55–2.88)	0.478
**Oscillation duration 3.2 km/h**	36.55 (2.03)	(35.89–37.21)	36.61 (2.24)	(35.88–37.34)	−0.06 (−1.95–1.83)	0.721
**Oscillation duration 4 km/h**	37.14 (2.02)	(36.48–37.80)	37.08 (2.29)	(36.33–37.83)	0.06 (−1.53–1.66)	0.635
**Oscillation duration 4.8 km/h**	37.91 (1.80)	(37.32–38.50)	37.84 (1.89)	(37.22–38.46)	0.07 (−1.38–1.51)	0.585
**Left foot bearing time normal speed**	63.62 (3.48)	(62.48–64.76)	63.45 (3.58)	(62.28–64.63)	0.17 (−1.99–2.32)	0.367
**Left foot bearing time 1.6 km/h**	61.88 (3.68)	(60.67–63.08)	63.53 (4.41)	(62.09–64.97)	−1.65 (−7.56–4.25)	0.002 *
**Left foot bearing time 2.4 km/h**	62.93 (2.43)	(62.13–63.72)	63.06 (2.79)	(62.14–63.97)	−0.13 (−3.64–3.38)	0.667
**Left foot bearing time 3.2 km/h**	62.12 (3.62)	(60.94–63.30)	61.99 (2.43)	(61.20–62.79)	0.13 (−3.88–4.13)	0.715
**Left foot bearing time 4 km/h**	60.85 (2.42)	(60.06–61.64)	60.87 (2.60)	(60.02–61.72)	−0.02 (−2.06–2.02)	0.918
**Left foot bearing time 4.8 km/h**	59.84 (2.23)	(59.11–60.57)	59.98 (2.40)	(59.20–60.77)	−0.14 (−2.12–1.84)	0.403
**Right foot bearing time normal speed**	63.72 (2.75)	(62.82–64.62)	63.80 (2.64)	(62.93–64.66)	−0.08 (−2.82–2.66)	0.730
**Right foot bearing time 1.6 km/h**	62.90 (3.43)	(61.78–64.03)	62.99 (4.50)	(61.51–64.43)	−0.08 (−5.98–5.82)	0.873
**Right foot bearing time 2.4 km/h**	62.91 (2.89)	(61.96–63.85)	63.08 (3.56)	(61.92–64.25)	−0.18 (−3.64–3.28)	0.547
**Right foot bearing time 3.2 km/h**	61.75 (2.10)	(61.06–62.44)	61.58 (2.65)	(60.71–62.44)	0.17 (−2.46–2.81)	0.442
**Right foot bearing time 4 km/h**	61.12 (2.27)	(60.38–61.87)	61.20 (2.48)	(60.39–62.01)	−0.08 (−2.24–2.09)	0.686
**Right foot bearing time 4.8 km/h**	60.34 (2.02)	(59.67–61.00)	60.27 (1.96)	(59.63–60.91)	0.07 (−1.74–1.88)	0.670
**Left foot swing time normal speed**	34.37 (3.54)	(33.21–35.53)	34.54 (3.42)	(33.33–35.57)	−0.08 (−2.33–2.18)	0.698
**Left foot swing time 1.6 km/h**	37.02 (3.33)	(35.93–38.11)	35.29 (4.24)	(33.90–36.62)	1.73 (−3.33–6.78)	0.000*
**Left foot swing time 2.4 km/h**	35.65 (2.43)	(34.85–36.45)	35.46 (2.91)	(34.51–36.41)	0.19 (−3.28–3.67)	0.516
**Left foot swing time 3.2 km/h**	36.51 (2.40)	(35.72–37.29)	36.38 (2.44)	(35.58–37.18)	0.13 (−1.87–2.14)	0.443
**Left foot swing time 4 km/h**	37.29 (2.42)	(36.49–38.08)	37.28 (2.59)	(36.43–38.12)	0.01 (−2.04–2.05)	0.961
**Left foot swing time 4.8 km/h**	38.16 (2.20)	(37.44–38.87)	38.03 (2.37)	(37.25–38.80)	0.13 (−1.89–2.14)	0.456
**Right foot swing time normal speed**	34.18 (2.71)	(33.30–35.07)	34.13 (2.60)	(33.28–34.98)	0.05 (−2.06–2.17)	0.771
**Right foot swing time 1.6 km/h**	35.81 (3.60)	(34.64–36.99)	35.83 (4.70)	(34.30–37.37)	−0.02 (−6.35–6.31)	0.971
**Right foot swing time 2.4 km/h**	35.65 (2.92)	(34.69–36.60)	35.57 (3.38)	(34.46–36.68)	0.08 (−3.01–3.16)	0.769
**Right foot swing time 3.2 km/h**	36.59 (2.14)	(35.89–37.29)	36.84 (2.63)	(35.98–37.70)	−0.25 (−2.83–2.34)	0.273
**Right foot swing time 4 km/h**	37.01 (2.27)	(36.27–37.75)	36.95 (2.48)	(36.13–37.76)	0.07 (−2.10–2.23)	0.724
**Right foot swing time 4.8 km/h**	37.62 (2.01)	(37.01–38.33)	37.72 (1.94)	(37.09–38.36)	−0.05 (−1.89–1.78)	0.733

Abbreviation: km/h (kilometers per hour); CI (confidence interval); SD (standard deviation); * (paired t-test significant differences, *p* < 0.05): LoA (limit of agreement).
